# The Association of Sleep Duration with Breakfast Patterns and Snack Behaviors among Chinese Children Aged 6 to 17 Years: Chinese National Nutrition and Health Surveillance 2010–2012

**DOI:** 10.3390/nu14112247

**Published:** 2022-05-27

**Authors:** Ailing Liu, Jing Fan, Caicui Ding, Fan Yuan, Weiyan Gong, Yan Zhang, Chao Song, Ying Zhou, Gangqiang Ding

**Affiliations:** 1 National Institute for Nutrition and Health, Chinese Center for Disease Control and Prevention, Beijing 100050, China; liual@ninh.chinacdc.cn (A.L.); jing_zwtcheroyl@163.com (J.F.); dingcc@ninh.chinacdc.cn (C.D.); yuanfan@ninh.chinacdc.cn (F.Y.); gongwy@ninh.chinacdc.cn (W.G.); zhangyan@ninh.chinacdc.cn (Y.Z.); songchao@ninh.chinacdc.cn (C.S.); zhouying@ninh.chinacdc.cn (Y.Z.); 2 Key Laboratory of Trace Element Nutrition, National Health Commission of the People’s Republic of China, Beijing 100050, China

**Keywords:** sleep duration, breakfast, snacks, eating behaviors, children

## Abstract

A significant increase in the prevalence of short sleep among children has been observed. Short sleep may be associated with unhealthy breakfast and snacking behaviors. The purpose of the current study was to explore the associations of short sleep with breakfast and snacking behaviors among children. Data were obtained from the 2010–2012 China National Nutrition and Health Surveillance (CNNHS). A total of 5254 children aged 6 to 17 years were included. Sleep duration was classified into three categories: moderate sleep, slightly short sleep, and severely short sleep. Breakfast behaviors included skipping breakfast, food diversity, intake of energy and macronutrients, and their proportion of daily total intake. Snack behaviors included snack consumption rate/frequency, types, intake of energy and macronutrients, and proportion of daily total intake. Multiple linear regression and multivariate logistic regression were used for analysis, with models adjusted for the potential effects of gender, age, region, and family income level. The bootstrapping method was used to calculate the 95% confidence intervals of the model statistics. Results showed that slightly short sleep (OR = 1.15, 95%CI: 1.00, 1.33)) and severely short sleep (OR = 1.36, 95%CI: 1.04, 1.77) was related to higher rates of skipping breakfast compared to moderate sleep. Severely short sleep was associated with higher energy (β = 28.44, 95%CI: 31.97, 44.70), carbohydrate (β = 6.62, 95%CI: 8.29, 8.84) and protein (β = 1.17, 95%CI: 1.44, 1.70) intake at breakfast and breakfast accounted for a higher proportion of total daily energy (β = 1.39, 95%CI: 1.48, 2.52), protein (β = 2.26, 95%CI: 3.16, 5.84) and carbohydrate (β = 0.83, 95%CI: 0.07, 3.41). Severely short sleep was associated with higher energy (β = 27.4, 95%CI: 18.64, 69.41), protein (β = 0.8, 95%CI: 0.48, 2.40), and fat (β = 1.40, 95%CI: 1.21, 3.16) intake at snacks and snacks accounted for a higher proportion of total daily protein intake (β = 1.23, 95%CI: 0.71, 3.58) and fat intake (β = 2.74, 95%CI: 3.13, 6.09). Slightly short sleep was associated with higher energy (β = 7.28, 95%CI: 0.15, 28.13) and carbohydrate (β = 1.67, 95%CI: 0.86, 5.73) intake at snacks and snacks accounted for a higher proportion of total daily carbohydrate intake. Children with severely short sleep were more likely to choose sugar-sweetened beverages (SSBs) as snacks (16.5%) and intake them more frequently, at a daily consumption of 204.7 g and 26.7 g per night. Overall, short sleep was associated with unhealthy breakfast patterns and snack behaviors among children. Children with short sleep had higher intake of energy and macronutrients at breakfast and snacks compared with those with moderate sleep. Promoting adequate sleep among children may have a positive effect on developing healthy eating behaviors.

## 1. Introduction

Sleep is the foundation of early childhood development. Adequate sleep plays an important role in promoting the growth and development of children and strengthening the immune system. Sleep-related issues in children have received more attention in recent years. This is against the fact that reports and studies from many countries show that children are experiencing a decrease in sleep duration and higher rates of sleep deprivation [1,2]. In 2015, the Youth Risk Behavior Surveys (YRBSs) revealed that 57.8% of American children in grades 6 to 8 and 72.2% in grades 9 to 12 suffered from insufficient sleep (insufficient sleep: sleep duration < 9 h/d for ages 6 to 12 and sleep duration < 8 h/d for ages 13 to 18) [1]. The Spanish national survey reveals that the prevalence of short sleep among children aged 2 to 14 years increased from 29.8% to 44.7% between 1987 and 2011 (short sleep: <11 h/d for children under 5 years, <10 h/d for children aged 5 to 10 years, and <9 h/d for children over 10 years), with an average decrease in sleep duration by 20 min [3].The China National Nutrition and Health Surveillance Report (2010–2013) shows that the average daily sleep duration was 8.9 h and 7.9 h for children aged 6 to 12 years and children aged 13 to 17 years, respectively, and decreased by 0.2 h and 0.6 h compared to 2002, respectively [4].The Healthy China Initiative (2019–2030) recommends that primary school students should get at least 10 h of sleep per day, and junior and senior high school students should get 9 and 8 h sleep, respectively, but Chinese children have not yet achieved the required amount [5].

The significant association of short sleep with obesity [6], diabetes [7,8], non-alcoholic fatty liver [9], and cardiovascular disease [10,11] have been reported, which maybe partly due to those children with short sleep are often accompanied by unhealthy eating behaviors, such as skipping breakfast, eating monotonous foods, snacking frequently and choosing unhealthy foods. A systematic review demonstrates that children eating breakfast every day are more likely to have higher dietary quality and lower body mass index (BMI) [12]. Studies have also shown that skipping breakfast was associated with a higher risk of cardiovascular disease in children [13,14] and was likely to persist in adulthood among children skipping breakfast [15], while skipping breakfast in adults could also be associated with high BMI and central obesity [16]. The Australian National Nutrition and Physical Activity Survey 2011–2012 showed that one of the attributes to skipping breakfast was sleep deprivation (ratio of actual sleep to adequate sleep < 0.75) [17]. A survey of teenagers also showed that adequate sleep (6–8 h per night) was associated with eating breakfast every day [18]. In the limited research on the relationship between short sleep and breakfast, skipping breakfast has received more attention, while other breakfast behaviors have not received sufficient attention. For example, do the children with short sleep consume more calories at breakfast or not? Do children with short sleep have a lower food diversity at breakfast or not? These questions are based on the mechanisms by which short sleep affects levels of appetite-regulating hormones and adipokines. Abnormal secretion of these hormones can cause increased signals of hunger or dissatisfaction, which in turn leads to increased energy intake and altered food choices in children [19]. Snacks often include many ultra-processed foods high in energy and low in nutrient density, and long-term consumption of these foods is harmful. Sugar-sweetened beverage (SSBs), for example, are very common snacks. Children with short sleep have reported higher consumption of SSBs compared to those with moderate sleep [20,21].

To summarize, considering that adequate sleep during childhood plays an important role in the development and maintenance of healthy eating behaviors and the prevention of disease in adulthood, it is necessary to pay attention to the association of short sleep with breakfast and snacking behaviors. However, few studies are available on this, especially for Chinese children. Moreover, studies focusing on skipping breakfast and snack intake/frequency are predominant, with less attention paid to other breakfast and snacking behaviors. Therefore, the current study used the China National Nutrition and Health Surveillance (CNNHS) to explore the association of short sleep with breakfast and snacking behaviors.

## 2. Materials and Methods

### 2.1. Data Source

Data came from the CNNHS in 2010–2012. The target population was the resident population of the sampled households in 31 provinces, autonomous regions, and municipalities directly under the Central Government (excluding Taiwan, Hong Kong, and Macao). For residents aged 6 years and above, a stratified multi-stage whole-group sampling method was employed to recruit subjects. China’s county-level administrative units were categorized by level and type of economic development into large cities, small and medium-sized cities, ordinary rural areas, and poor rural areas. Overall, 34 monitoring sites (districts/counties) were selected from large cities, 41 from small and medium-sized cities, 45 from ordinary rural areas, and 30 from poor rural areas. A total of 6 residential (village) committees were selected from each monitoring site, and 75 households were selected from each sampled committee by simple random sampling method. To ensure the required sample size for children aged 6 to 17 years, the sample area was appropriately supplemented with the number of children surveyed in this population.

### 2.2. Subjects and Ethics

After excluding the children with missing basic personal information, or less than 1 day of dietary records, or daily energy intake less than 800 kcal (3345.6 KJ) or more than 5000 kcal (20,910 KJ) per day, 5254 children were eventually included in the current analysis. This study was approved by the ethics review committee of the National Institute for Nutrition and Health, Chinese Center for Disease Control and Prevention (No. 2013–018), and all participants and their guardians signed the informed consent.

### 2.3. Data Collection

The dietary collection was conducted by trained investigators in household visits and consisted of a 3-day consecutive (2 weekdays and 1 weekend-day) 24-h dietary recall and household weighing method. Children were asked about their food intake during the past 24 h before each household visit, including the type and weight of each food item, cooking method, and eating occasion. The food pictures and models were used to facilitate a more accurate and complete recall. When children were unable to recall accurately, their guardians, the person who prepared the cuisine, or the person jointly eating the meal were interviewed to complete the dietary recall. The consumption of edible oils, salt, sugar, and other condiments was collected by the household weighing method.

Sleep duration was collected by an interview-administrated questionnaire. All the children were asked the question “how long did you usually sleep per day last week?”. The children younger than 10 years old finished the question with the help of their guardians. 

### 2.4. Variables

#### 2.4.1. Sleep Duration

Based on the National Sleep Foundation’s (NSF) recommendations, children aged 6–13 years with more than 11 h of sleep, 9 to 11 h, 7 h or more but less than 9 h, and less than 7 h were classified as long sleep, moderate sleep, slightly short sleep, and severely short sleep, respectively. Children aged 14 to 17 years with more than 10 h of sleep, 8 to 10 h, 6 h or more but less than 8 h, and less than 6 h were classified as a long sleep, moderate sleep, slightly short sleep, and severely short sleep, respectively. Only 30 children (0.5%) in the current study were classified as long sleep and were therefore deleted for further analysis.

#### 2.4.2. Breakfast Patterns

Breakfast patterns include the following four variables: (1) Skipping rate: a dichotomous variable, categorized into <3 times and 3 times during the 3-day dietary recall. (2) Food diversity: Chinese Dietary Guidelines classifies food types into four major categories: cereals and potatoes and mixed beans; animal foods; milk products, soybeans products and nuts; vegetables and fruits. Food types at breakfast including three or more of these four categories were considered as meeting the food diversity criteria and vice versa. (3) Energy and macronutrients intake. (4) The proportion of energy and macronutrient intake in breakfast to the total daily intake.

#### 2.4.3. Snacks Behaviors

According to the Chinese Dietary Guideline, the snack was defined as food consumed on any occasion other than traditional meals (breakfast, lunch, and dinner). Snack behaviors included the following three variables: (1) Frequency: divided into ≥1 time/day and <1 time/day. (2) Snack types: The types of snack consumption were ranked by consumption frequency and by the amount consumed, respectively, in a complete 24 h period and in the period between dinner and breakfast in the subsequent day. In the former ranking, the occurrence of a snack food item in the dietary record was counted as one occasion, regardless of the amount consumed, and the top 5 food items were summed up as the 5 most frequently consumed. The latter ranking refers to the top 5 food items with the highest amount of snack consumption by summing up the amount of consumption of each snack food item in the dietary records. (3) Energy and macronutrients supply ratio: the proportion of energy and macronutrients from snacks to total energy intake throughout the day and the proportion from snacks to total intake in the period between dinner and breakfast in the subsequent day.

#### 2.4.4. Demographic Characteristics

Age was divided into two groups (6–12 years and 13–17 years). Large cities and small and medium-sized cities were combined into urban areas, and ordinary rural areas and poor rural areas were combined into rural areas. Family income level was divided into four groups (low, medium, high, and unclear) according to the 2009 National Net Income Register of the National Bureau of Statistics.

### 2.5. Statistical Analysis

All data were collated and analyzed in the Statistical Analysis System(SAS) 9.4 software (SAS Institute Inc., Cary, NC, USA) and a two-sided *p* < 0.05 was considered statistically significant. Continuous variables were expressed as means, and categorical variables were expressed as percentages. Multivariate logistic regression were used to analyze the association of sleep duration with breakfast pattern and snack behavior, where the independent variable was sleep duration (moderate sleep = 0, slightly short sleep = 1, and severely short sleep = 2), dependent variable was skipping breakfast ( no = 0, yes = 1), breakfast food diversity (no = 0, yes = 1), snack consumption (no = 0, yes = 1), snack consumption frequency (<1 time/day = 0, ≥1 time/day = 1), post-dinner snacks consumption (no = 0, yes = 1), respectively, adjusting the potential influence of gender, age, region, and family income level. Multiple linear regression was used to analyze the association of sleep duration with energy and macronutrients intake of breakfast and snacks, where sleep duration was an independent variable (moderate sleep = 0, slightly short sleep = 1, and severely short sleep = 2), dependent variable was energy intake from breakfast, carbohydrate intake form breakfast, fat intake form breakfast, proportion of energy intake from breakfast to total daily intake, proportion of protein intake from breakfast to total daily intake, proportion of carbohydrate intake from breakfast to total daily intake, proportion of fat intake from breakfast to total daily intake, respectively, adjusting the potential influence of gender, age, region, and family income level. The same multiple linear regression was used to analyze the association of sleep duration with energy and macronutrients intake of breakfast and snack, and the proportion of energy and macronutrients intake from sacks to total daily intake.

The bootstrapping method allows for a fuller exploitation of the information carried by the original observed sample data as well as free from the reliance on distributional assumptions of traditional statistical methods, which was used to recalculate the confidence intervals of linear regression and logistic regression statistics in this study (Reps = 10,000). This analysis was based on the use of R language.

## 3. Results

### 3.1. Basic Characteristics

Of the 5254 children, 32.7% were slightly sleep-deprived and 5.4% severely sleep-deprived. Sleep duration varied by age, region, and family income level, with all differences statistically significant. The prevalence of severely sleep-deprived children was higher among the elderly, rural, and low-income families, at 12.7%, 6.6%, and 6.1%, respectively (Table 1). 

### 3.2. Breakfast Patterns

The rate of skipping breakfast among children was 24.0%, and 61.0% of children met breakfast dietary diversity. Both the rate of skipping breakfast and the rate of breakfast dietary diversity varied among children with different sleep durations (Table 2). Adjusting for the influences of gender, age, region, and family income level, children with slightly short sleep (OR = 1.15, 95% CI: 1.00, 1.33) and with severely short sleep (OR = 1.36, 95% CI: 1.03, 1.77) had a higher rate of skipping breakfast compared to children with moderate sleep. No significant association was found between breakfast food diversity and sleep duration (Table 3).

As shown in Table 2, the intake of energy, carbohydrate, protein, and fat from breakfast was 344.7 kcal, 58.3 g, 11.2 g, and 7.4 g, respectively. The ratio of them to total daily intake was 24.1%, 25.0%, 21.7%, and 12.6%, respectively. Adjusting for the effects of gender, age, region, and family income level, severely short sleep was associated with higher energy (β = 28.44, 95%CI: 31.97, 44.70), carbohydrate (β = 6.62, 95%CI: 8.29, 8.84) and protein (β = 1.17, 95%CI:1.44, 1.70) intake at breakfast. Breakfast accounted for a higher proportion of total daily energy intake (β = 1.39, 95%CI: 1.48, 2.52), total daily protein intake (β = 2.26, 95%CI: 3.16, 5.84) and total daily carbohydrate (β = 0.83, 95%CI: 0.07, 3.41) for children with severely short sleep compared to those with moderate sleep, while breakfast accounted for a lower proportion of total daily carbohydrate intake for children with slightly short sleep (β = −0.74, 95%CI: −1.43, −0.75). No significant association was found between the ratio of fat to total daily intake and sleep duration (Table 4).

As shown in Table 2 and Table 3, 53.2% of children consumed snacks, which was lower among children with slightly short sleep (OR = 0.78, 95%CI: 0.68–0.89) and severely short sleep (OR = 0.63, 95%CI: 0.48–0.82) compared to those with moderate sleep after adjusting for the influence factors. The rate of consuming snacks at least once per day was 28.3%, and no significant difference among the three sleep groups was found. The average daily snacks energy supply ratio was 9.4%, and the post-dinner snacks energy supply ratio was 5.2% among all snack consumers. Adjusting for the effects of gender, age, region, and family income level, severely short sleep was associated with higher energy (β = 27.4, 95%CI: 18.64, 69.41), protein (β = 0.8, 95%CI: 0.48, 2.40), and fat (β = 1.40, 95%CI: 1.21, 3.16) intake at snacks, while slightly short sleep was associated with higher energy (β = 7.28, 95%CI: 0.15, 28.13) and carbohydrate (β = 1.67, 95%CI: 0.86, 5.73) intake at snacks. Snacks accounted for a higher proportion of total daily energy intake (β = 1.17,95%CI: 0.69, 3.03), total daily protein intake (β = 1.23, 95%CI: 0.71, 3.58) and total daily fat intake (β = 2.74, 95%CI: 3.13, 6.09) for children with severely short sleep compared to those with moderate sleep, while a higher proportion of total daily carbohydrate intake (β = 1.02, 95%CI: 0.78, 2.83) among children with slightly short sleep duration (Table 5).

**Figure 1:**
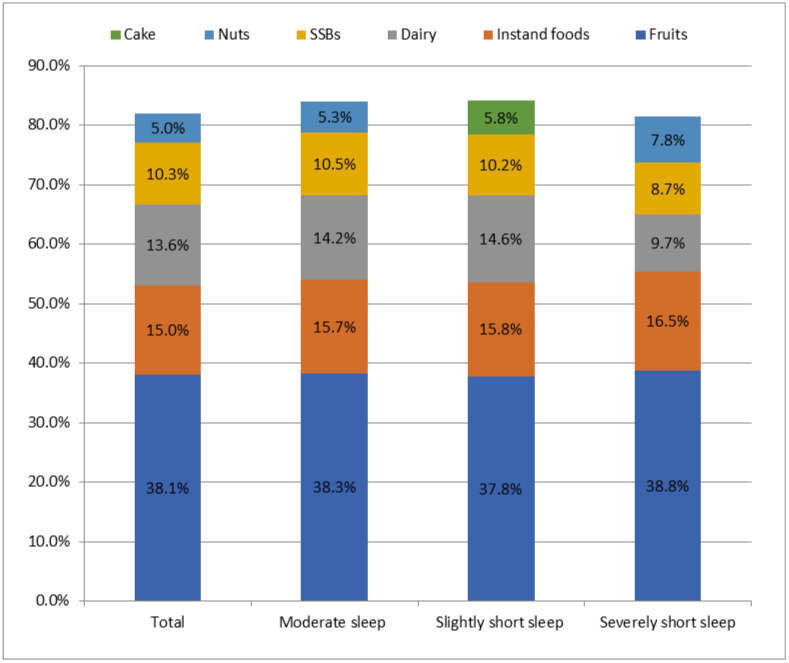
The top 5 snack types with highest consumption rate in children with different sleep durations.

**Figure 2:**
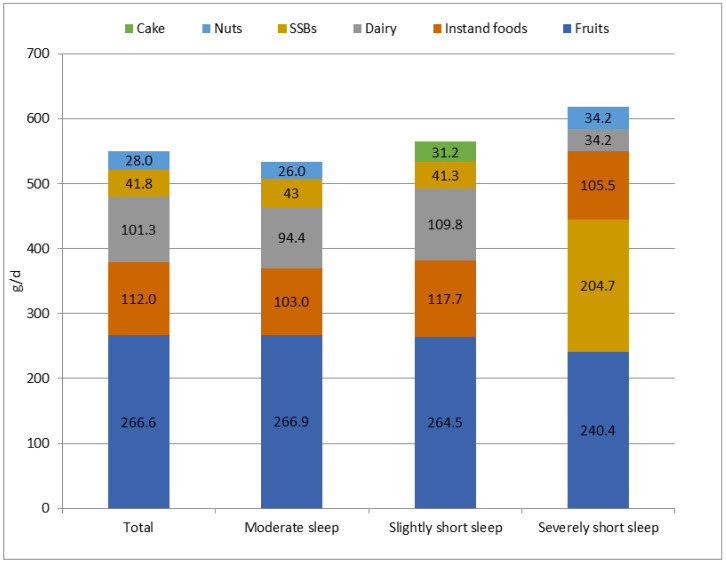
The top 5 snack types with highest daily intake in children with different sleep durations.

**Figure 3:**
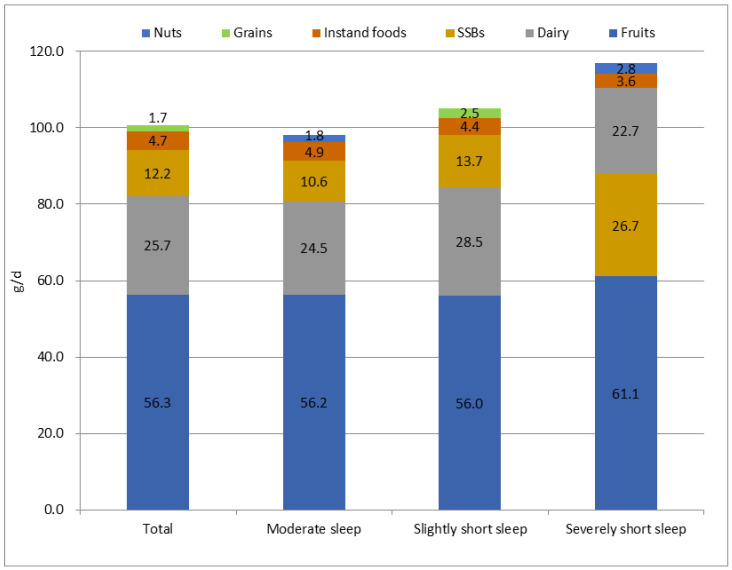
The top 5 snack types with highest daily intake at night in children with different sleep durations.

As shown in Figure 1, Figure 2 and Figure 3, the most frequently consumed snack in all three sleep groups was fruit, followed by instant food in moderate sleep group and slightly short sleep group and SBBs (16.5%) in severely short sleep group. The snack with the highest intake during the whole day and at night in the three group was fruits, followed by instant food in moderate sleep group and slightly short sleep group and SBBs (204.7 g/d, 26.7 g/d, respectively) in severely short sleep group.

## 4. Discussion

The current study revealed the associations of sleep duration with breakfast patterns and snack behaviors, most of them being considered unhealthy. Previous studies had suggested that short sleep might be related to the intake of food groups, energy, and macronutrients. Pérez-Farinós, N. et al. [21] found that short sleep (<9.9 h/day) was associated with high cereal intake (*OR* = 1.14, 95% *CI*: 1.06, 1.23) in 6287 Spain children aged 6–9 years in 2011. Hart, C. N. et al. [3] conducted a randomized controlled trial in children aged 8–11 years and showed that a reduction in sleep duration of 2 h and 21 min per night on average for one week was associated with an increase in average daily energy intake of 134 kcal. Coronado Ferrer, S. et al. [22] found that one additional hour of sleep was associated with increased fat intake (*β* = −11.11, 95% *CI*: −19.44, −2.78, *p* = 0.0069) compared to short sleep (<9 h) in Spanish school-age children. However, most of these studies explored differences in whole-day food and nutrients intakes at different sleep durations. Limited data exists concerning the relationship between sleep and energy and macronutrients in a particular single eating occasion (such as breakfast and snacks). Nedeltcheva, A. V. et al. [23] found that short sleep (5.5 h) was associated with significantly higher total energy intake among participants aged 34–49 years with sedentary behaviors (*p* = 0.04), and the main contributor to this difference was snacks (*p* = 0.04) rather than meals (*p* = 0.49). Kant, A. K., & Graubard, B. I. [24] analyzed food consumption data from NHANES 2005–2010 survey and also found that participants aged 20 years and older with short sleep obtained higher energy from snacks than from main meals (*p* < 0.0004), and relative to those with average sleep duration, a higher percentage of those with short sleep reported received ≥50% more energy (*p* = 0.002). The current study identified similar findings in children to those of Nedeltcheva, A. V. et al. [23] and Kant, A. K., & Graubard, B. I. [24], that is, children with short sleep had higher levels of energy, carbohydrate, fat and protein intake at snacks. There are some studies suggested that children with short sleep were more likely to skip breakfast. Konstantinos, D. T. et al. [25] investigated the sleep duration and eating habits of 177,091 Greek children aged 8–17 years and revealed that children with short sleep (<9 h/day for children and <8 h/day for adolescents) were 1.30 times more likely to skip breakfast than children with adequate sleep (*p* < 0.05). A study of 1033 primary school children in grades 1 to 6 in Ecuador showed that children with enough sleep (≥ 9 h) were more likely to eat breakfast every day (*OR* = 1.44, 95% *CI*: 1.01, 2.08) [26]. In this regard, Al-Hazzaa, H.M. et al. [26] suggested that children with short sleep seldom have time to eat breakfast at home before school, especially now that school starts early, and that regular adequate sleep and access to breakfast every day may also reflect a more stable family structure and a high level of supervision with an organized family. The mechanisms of the relationship of sleep duration and food intake had been reported in previous studies, which implied that short sleep might influence food and energy intake by altering the human physiological cycle and levels of hunger and satiety hormones (leptin and ghrelin), as well as by reducing insulin sensitivity [27,28]. Breakfast being the first meal after waking up from the longest dormancy of the day, the effects of sleep deprivation may be more noticeable compared to lunch and dinner. 

The current study also found that children with severely short sleep had higher levels of SSBs intake, which is similar to some previous studies. Mozaffarian, N. et al. [29] found that sleep deprivation (6–10 years: <10 h; 10 years and older: <9 h) was a risk factor for higher SSBs intake (*OR* = 1.12, 95%*CI*: 1.04, 1.20) based on a national cross-sectional survey of 14,274 Iranian children aged 6–18 years. In a cross-sectional study of 5873 children aged 9–11 years, Chaput, J. P. et al. [30] found that children who consumed SSBs at least once a day were 12 min shorter in sleep duration per night compared to those who consumed them less than once a week, and children who could get enough sleep duration (9 to 11 h) were less likely to report consuming SSBs at least once a week compared to those who slept less than 9 h (*OR* = 0.79, 95%*CI*: 0.71, 0.88). Short sleep as a risk factor of adverse changes to metabolic hormones in the body was associated with an increased appetite for sweet foods [31]. We also found that children with severely short sleep had higher SSBs consumption levels at night. Little attention has been paid to what children eat at night (after dinner and before bedtime), and we hypothesize that children with short sleep may have a higher intake of energy or unhealthy foods during this period, which was indeed observed in the current study. However, we were not able to retrieve relevant kinds of literature and this relationship needs to be further confirmed by further studies.

There are certain limitations to this study. Firstly, the measurement of sleep is complex, as it includes not only sleep duration but also sleep onset, sleep midpoint, and sleep quality [32,33], and the current survey was only able to obtain sleep duration and the proportion of excessive sleep was quite low, which may have affected the results. Secondly, sleep duration was self-reported, making it less accurate than objective measures. Thirdly, children usually grow up under the supervision of their parents or other guardians, and the influence of these guardians on children’s eating behavior may not be negligible, which suggested that the characteristics of their guardians should also be included. Finally, the current study was cross-sectional and causal associations of breakfast patterns and snack behaviors with sleep duration could not be established. However, this study still provides epidemiological evidence in a larger population of Chinese children that short sleep was associated with skipping breakfast, high energy, carbohydrate, fat and protein intake at breakfast and snacks, and high SSBs intake levels, where the use of bootstrapping method also confirms the levels of significance.

## 5. Conclusions

Short sleep was associated with skipping breakfast, high energy, carbohydrate and protein intake at breakfast and snacks, and high SSBs intake levels.Ensuring adequate sleep for children is of great significance in promoting the development of healthy breakfast patterns and snack behaviors. 

## Figures and Tables

**Table 1:** Sleep duration of children with different characteristics.

Variables	Total	Moderate Sleep	Slightly Short Sleep	Severely Short Sleep	*p*
	n	n (%)	n (%)	n (%)	
Total	5254	3255 (62.0)	1716 (32.7)	283 (5.4)	
Gender					
Male	2817	1742 (62.0)	912 (32.4)	163 (5.8)	0.3738
Female	2437	1513 (62.1)	804 (33.0)	120 (4.9)	
Age (years)					
6–12	3573	2149 (60.2)	1356 (38.0)	68 (1.9)	<0.0001
13–17	1681	1106 (66.0)	360 (21.4)	215 (12.8)	
Region					
Urban	2162	1282 (59.3)	802 (37.1)	78 (3.6)	<0.0001
Rural	3092	1973 (63.8)	914 (29.6)	205 (6.6)	
Income level					
Low	3060	1927 (63.1)	947 (31.0)	186 (6.1)	0.0032
Middle	1533	937 (61.1)	521 (34.0)	75 (4.9)	
High	393	235 (60.0)	148 (37.7)	10 (2.5)	
Unknown	268	156 (58.2)	100 (37.3)	12 (4.5)	

Moderate sleep: 9–11 h/d for 6–13 years children, 8–10 h/d for 14–17 years children; slightly short sleep: 7 h/d or more but less than 9 h/d for 6–13 years children, 6 h/d or more but less than 8 h/d for 14–17 years children; severely short sleepless than 7 h/d for 6–13 years children, less than 6/d for 14–17 years children.

**Table 2:** Breakfast patterns of children with different sleep duration.

	Total	Moderate Sleep	Slightly Short Sleep	Severely Short Sleep	*p*
Breakfast pattern					
Skipping breakfast (N, %)	1263 (24.0)	753 (23.1)	414 (24.1)	96 (33.9)	0.0018
Breakfast food diversity (N, %)	3226 (61.0)	1987 (61.0)	1039 (60.6)	181 (64.0)	0.7008
Intake from breakfast					
Energy (kcal/d, % *)	344.7 (24.1)	343.4 (24.1)	339.9 (24.9)	392.4 (24.7)	0.0357
Carbohydrate (g/d, % *)	58.3 (25.0)	58.4 (25.1)	56.4 (24.9)	69.7 (24.9)	0.0748
Protein (g/d, % *)	11.2 (21.7)	11.2 (21.6)	11.2 (21.6)	12.8 (23.2)	0.0214
Fat (g/d, % *)	7.4 (12.6)	7.2 (12.5)	7.7 (13.0)	7.0 (12.5)	0.2100
Snack behavior					
Rate of snack consumption (N, %)	2794 (53.2)	1794 (55.1)	890 (51.9)	110 (38.9)	<0.0001
Rate of snack consumption ≥ 1 times/d (N, %)	1487 (28.3)	938 (28.8)	494 (28.8)	55 (19.4)	0.0312
Rate of post-dinner snacks consumption (N, %)	1688 (32.1)	1066 (32.3)	561 (32.7)	61 (21.6)	0.0127
All-day snacks					
Energy (kcal/d, % ^#^)	166.4 (9.4)	161.9 (9.2)	173.9 (9.6)	180.5 (9.4)	0.0688
Carbohydrate (g/d, % ^#^)	27.7 (12.3)	29.0 (11.8)	29.2 (13.5)	28.8 (11.3)	0.0791
Protein (g/d, % ^#^)	4.2 (7.5)	5.0 (7.3)	4.4 (7.8)	4.6 (7.5)	0.1202
Fat (g/d, % ^#^)	5.2 (8.1)	7.2 (7.9)	8.3 (8.3)	6.1 (9.9)	0.1815
Post-dinner snacks					
Energy (kcal/d, % ^#^)	92.3 (5.2)	91.2 (5.2)	92.4 (5.1)	110.3 (5.9)	0.3391
Carbohydrate (g/d, % ^#^)	15.5 (7.1)	15.0 (6.6)	16.0 (7.8)	19.1 (8.1)	0.0670
Protein (g/d, % ^#^)	2.3 (4.2)	2.4 (4.2)	2.7 (4.0)	4.0 (4.6)	0.7753
Fat (g/d, % ^#^)	2.9 (4.4)	3.0 (4.5)	2.2 (4.1)	6.9 (5.6)	0.5196

Moderate sleep: 9–11 h/d for 6–13 years children, 8–10 h/d for 14–17 years children; slightly short sleep: 7 h/d or more but less than 9 h/d for 6–13 years children, 6 h/d or more but less than 8 h/d for 14–17 years children; severely short sleepless than 7 h/d for 6–13 years children, less than 6/d for 14–17 years children. Skipping breakfast was defined as <3 times during the 3-day dietary recall. Breakfast food diversity was defined as consumption of three or more of the four food categories: cereals, potatoes and mixed beans; animal foods; milk and milk products, soybeans and nuts; vegetables and fruits. * Proportion of energy and macronutrients intakes from breakfast to total daily intake. ^#^ Proportion of energy and macronutrients intakes from snacks to total daily intake.

**Table 3:** Logistic regression of breakfast skipping and food diversity with sleep duration.

	Skipping Breakfast		Breakfast Food Diversity		Rate of Snack Consumption		Rate of Snack Consumption ≥ 1 Times/d		Rate of Post-Dinner Snacks CONSUMPTION	
	OR	95%CI	OR	95%CI	OR	95%CI	OR	95%CI	OR	95%CI
Original										
Moderate sleep	ref		ref		ref		ref		ref	
Slightly short sleep	1.15	(1.00, 1.33)	0.94	(0.83, 1.06)	0.78	(0.69, 0.89)	0.88	(0.78, 1.01)	0.9	(0.79, 1.02)
Severely short sleep	1.36	(1.04, 1.77)	1.29	(0.99, 1.67)	0.63	(0.49, 0.82)	0.78	(0.57, 1.07)	0.69	(0.51, 0.93)
Bootstrapping										
Moderate sleep	ref		ref		ref		ref		ref	
Slightly short sleep	1.15	(1.00, 1.33)	0.94	(0.84, 1.06)	0.78	(0.69, 0.89)	0.89	(0.78, 1.02)	0.90	(0.79, 1.03)
Severely short sleep	1.36	(1.03, 1.77)	1.29	(0.99, 1.67)	0.63	(0.48, 0.82)	0.78	(0.57, 1.06)	0.68	(0.50, 0.93)

Abbreviations: OR: odds ratio, CI: confidence interval. Moderate sleep: 9–11 h/d for 6–13 years children, 8–10 h/d for 14–17 years children; slightly short sleep: 7 h/d or more but less than 9 h/d for 6–13 years children, 6 h/d or more but less than 8 h/d for 14–17 years children; severely short sleepless than 7 h/d for 6–13 years children, less than 6/d for 14–17 years children. Skipping breakfast was defined as <3 times during the 3-day dietary recall. Breakfast food diversity was defined as consumption of three or more of the four food categories: cereals, potatoes and mixed beans; animal foods; milk and milk products, soybeans and nuts; vegetables and fruits.

**Table 4:** Linear regression between sleep duration and breakfast energy and macronutrient intake.

	Energy Intake		Carbohydrate Intake		Protein Intake		Fat Intake		Energy Ratio *		Carbohydrate Ratio *		Protein Ratio *		Fat Ratio *	
	β	95%CI	β	95%CI	β	95%CI	β	95%CI	β	95%CI	β	95%CI	β	95%CI	β	95%CI
Original																
Moderate sleep	ref		ref		ref		ref		ref		ref		ref		ref	
Slightly short sleep	1.04	(−9.95, 12.03)	−0.82	(−2.85, 1.21)	0.07	(−0.33, 0.46)	0.45	(0.00, 0.90)	−0.55	(−1.15, 0.05)	−0.74	(−1.45, −0.03)	−0.29	(−0.90, 0.33)	0.26	(−0.43, 0.95)
Severely short sleep	28.44	(4.58, 52.30)	6.62	(2.21, 11.02)	1.17	(0.31, 2.03)	−0.30	(−1.27, 0.66)	1.39	(0.09, 2.69)	0.83	(−0.71. 2.39)	2.26	(0.93, 3.60)	0.55	(−0.95. 2.06)
Bootstrapping																
Moderate sleep	ref		ref		ref		ref		ref		ref		ref		ref	
Slightly short sleep	1.04	(−9.01–13.12)	−0.82	(−3.81–0.42)	0.07	(−0.27–0.54)	0.45	(0.45–0.88)	−0.55	(−1.03, −0.49)	−0.74	(−1.43, −0.75)	−0.29	(−1.13, 0.04)	0.26	(−0.17, 1.23)
Severely short sleep	28.44	(31.97–44.70)	6.62	(8.29–8.84)	1.17	(1.44–1.70)	−0.30	(−1.62–0.39)	1.39	(1.48, 2.52)	0.83	(0.07, 3.41)	2.26	(3.16, 5.84)	0.55	(−0.41, 2.81)

Moderate sleep: 9–11 h/d for 6–13 years children, 8–10 h/d for 14–17 years children; slightly short sleep: 7 h/d or more but less than 9 h/d for 6–13 years children, 6 h/d or more but less than 8 h/d for 14–17 years children; severely short sleepless than 7 h/d for 6–13 years children, less than 6/d for 14–17 years children. Reference group: moderate sleep.* Proportion of energy and macronutrients intakes from breakfast to total daily intake. Models were adjusted for gender, age, region, and family income level. 3.3. Snack behaviors.

**Table 5:** Linear regression between sleep duration and energy and macronutrient intake from snacks.

	Energy Intake		Carbohydrate Intake		Protein Intake		Fat Intake		Energy Ratio *		Carbohydrate Ratio *		Protein Ratio *		Fat Ratio *	
	β	95%CI	β	95%CI	β	95%CI	β	95%CI	β	95%CI	β	95%CI	β	95%CI	β	95%CI
Original																
Moderate sleep	ref		ref		ref		ref		ref		ref		ref		ref	
Slightly short sleep	7.28	(−7.06, 21.62)	1.67	(−0.80, 4.13)	0.16	(−0.27, 0.60)	0.09	(−0.54, 0.72)	0.05	(−0.61, 0.71)	1.02	(−0.17, 2.21)	0.29	(−0.41, 0.99)	0.26	(−0.67, 1.20)
Severely short sleep	27.4	(−7.73, 62.01)	2.91	(−3.03, 8.85)	0.8	(−0.26, 1.85)	1.4	(−0.12, 2.92)	1.17	(−0.42, 2.76)	1.32	(−1.55, 4.20)	1.23	(−0.44, 2.90)	2.74	(0.48, 4.99)
Bootstrapping																
Moderate sleep	ref		ref		ref		ref		ref		ref		ref		ref	
Slightly short sleep	7.28	(0.15, 28.13)	1.67	(0.86, 5.73)	0.16	(−0.12, 0.82)	0.09	(−0.46, 0.83)	0.05	(−0.58, 0.76)	1.02	(0.78, 2.83)	0.29	(−0.12, 1.35)	0.26	(−0.39, 1.53)
Severely short sleep	27.4	(18.64, 69.41)	2.91	(−0.23, 12.28)	0.8	(0.48, 2.40)	1.4	(1.21, 3.16)	1.17	(0.69, 3.03)	1.32	(−0.16, 12.18)	1.23	(0.71, 3.58)	2.74	(3.13, 6.09)

Moderate sleep: 9–11 h/d for 6–13 years children, 8–10 h/d for 14–17 years children; slightly short sleep: 7 h/d or more but less than 9 h/d for 6–13 years children, 6 h/d or more but less than 8 h/d for 14–17 years children; severely short sleepless than 7 h/d for 6–13 years children, less than 6/d for 14–17 years children. Reference group: moderate sleep. * Proportion of energy and macronutrients intakes from snacks to total daily intake. Models were adjusted for gender, age, region, and family income level.

## Data Availability

The data presented in this study are non-public.
